# MyLabStocks: a web-application to manage molecular biology materials

**DOI:** 10.1002/yea.3008

**Published:** 2014-04-09

**Authors:** Florent Chuffart, Gaël Yvert

**Affiliations:** Laboratoire de Biologie Moléculaire de la Cellule, Ecole Normale Supérieure de Lyon, CNRS, Université de LyonFrance

**Keywords:** yeast strains, plasmids, oligonucleotides, software, laboratory management

## Abstract

Laboratory stocks are the hardware of research. They must be stored and managed with mimimum loss of material and information. Plasmids, oligonucleotides and strains are regularly exchanged between collaborators within and between laboratories. Managing and sharing information about every item is crucial for retrieval of reagents, for planning experiments and for reproducing past experimental results. We have developed a web-based application to manage stocks commonly used in a molecular biology laboratory. Its functionalities include user-defined privileges, visualization of plasmid maps directly from their sequence and the capacity to search items from fields of annotation or directly from a query sequence using BLAST. It is designed to handle records of plasmids, oligonucleotides, yeast strains, antibodies, pipettes and notebooks. Based on PHP/MySQL, it can easily be extended to handle other types of stocks and it can be installed on any server architecture. MyLabStocks is freely available from: https://forge.cbp.ens-lyon.fr/redmine/projects/mylabstocks under an open source licence.

## Introduction

Every molecular biology laboratory uses and stores thousands of biological products. Efficiently managing information about these products is crucial for day-to-day work. Minimally, researchers need to know what materials have been stored, where, when, by whom and for what purpose. They also need to access various types of information about every item: its source, its sequence, whether it had already been successfully used in past experiments and so on. Information about a reagent should be easy to update, and the data is to be shared among members of the laboratory. One difficulty is the high turnover of students and post-doctoral workers. It is often challenging to retrieve information about a reagent used by someone who left the laboratory long ago.

For this reason, many laboratories have developed their own solution. Sometimes, a minimal implementation is done by simply editing datasheets stored on a computer. A better solution is to use a database that can be archived, updated and searched. Ideally, installing this database on a server rather than a local computer allows all members of the laboratory to use it as 'clients'. Commercial database softwares, such as FileMaker, have sometimes been used because they offer a user-friendly interface to develop the database and display information (Nayler and Stamm, 1999). This solution is sometimes sufficient, but it requires to purchase the necessary number of licences so that all laboratory members can easily access data from various computers. Other companies offer storage services on the internet. This way, information about reagents can be stored and interrogated remotely from anywhere. However, these services are sometimes technically limited, e.g. if fields are predefined and can not be modified easily, or if information can not be searched with sequence analysis tools. In addition, uploading research data on a private external server may raise important issues about security and confidentiality. Research institutions therefore often recommend storing the database on an in-house server.

Dedicated commercial solutions, such as LabCollector (http://labcollector.com/), offer advanced possibilities. However, we consider that developing an open source alternative is important because of the following limitations of commercial softwares. First, users do not have the possibility to develop novel functionalities. Many biologists are familiar enough with programming to adapt database features to their needs. This contribution requires access to the software code. Secondly, commercial providers can not guarantee free financial cost in the long term. Once the data are stored in a given format, licences must be purchased to upgrade the software or to equip more computers. Finally, and perhaps more importantly, an open source solution provides total and guaranteed freedom of access to a vital organ of the laboratory. If maintenance by the provider is discontinued, or if it reaches a prohibitive price, users still have the possibility to access the data and code and they can look for alternative solutions. To our knowledge, there are currently two open source solutions dedicated to laboratory stocks management: LabStoRe (http://bioinformatics.org/phplabware/labwiki/index.php) and OpenFreezer (Olhovsky *et al*., 2011). They are both well adapted to enter, edit and search items based on annotations or storage locations. OpenFreezer offers advanced possibilities to enter details about specific reagents, such as plasmid maps. However, neither of these programs provide any dynamic functionality that can retrieve information directly from DNA or protein sequences. Plasmid maps displayed by OpenFreezer are produced from static text files that must be edited by users, and stocks can not be searched by homology to a query sequence.

We therefore have developed MyLabStocks, an open source software that stores information about laboratory stocks and uses sequence analysis tools to help users display, search and edit database records.

## Results and discussion

### Tables of laboratory material

**Figure 1:**
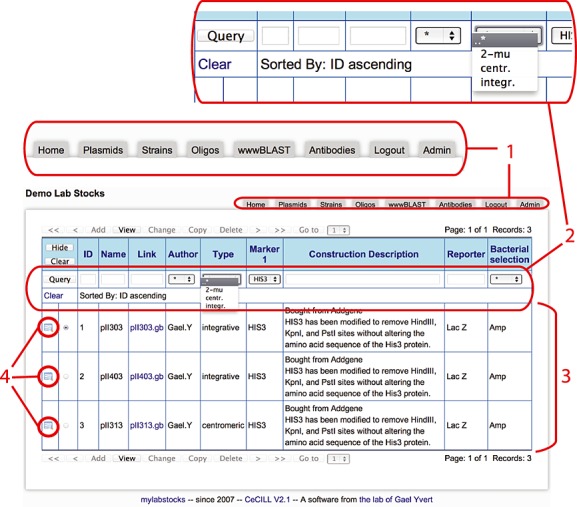
MyLabStocks Environment. Tabs in area 1 facilitate navigation through the various tables of materials. In this screenshot, the active table is the one storing plasmids information. Area 2 offers quick and easy tools to sort and search the table. All or selected items of the table are listed in area 3. For each item, an icon (4) provides a link to a page displaying detailed information on the item

**Figure 2:**
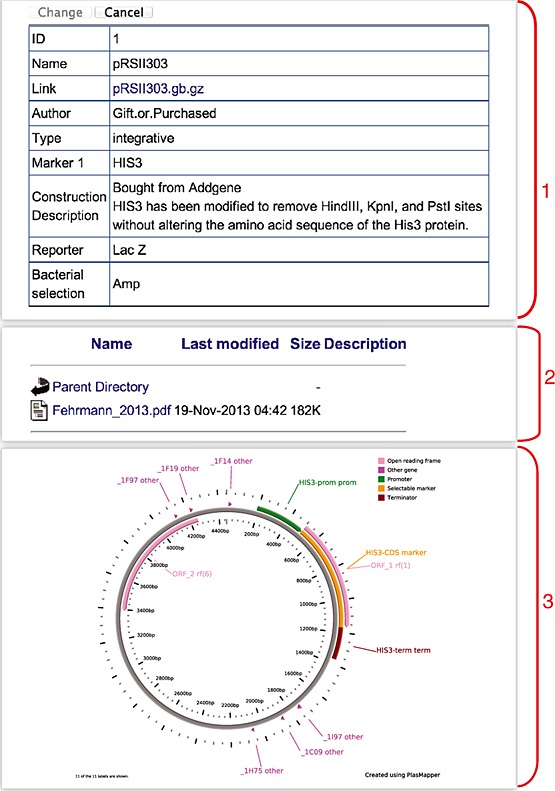
Detailed page of a laboratory item. MyLabStocks displays complete information on any item in a single page. The upper area (1) lists all annotations that were entered about the item. The following area (2) displays the content of the item's subdirectory, where files can be uploaded and retrieved. For plasmids, a third area (3) displays an image of the plasmid map, which is dynamically created from the sequence every time the page is viewed

In its current release, MyLabStocks handles the following types of material: plasmids, oligonucleotides, yeast strains, antibodies, pipettes and laboratory notebooks. For each type, a table stores information about every item in the laboratory. Table 1 summarizes the fields of each table. Tabs are located in page headers to help users navigate from one table to another. Once a table is displayed, users can sort items according to any field (Figure 1). The details of any record can be accessed in one click that produces an item-specific formatted page (Figure 2). This page displays the field's information as well as the content of the item's subdirectory. Any file containing additional information about the item can be uploaded in this subdirectory (when logged in add or edit mode, see below). For example, when entering a new antibody in the database, a user can upload in this directory the product datasheet from the commercial provider, a microscopy image he/she obtained with this antibody, a material and transfer agreement, or any other informative file. This way, any user can later download these documents directly from the item's page. For yeast strains, the item's page displays automatically the complete genotype of the strain by grouping information from all fields corresponding to genetic loci. For plasmids, MyLabStocks produces a plasmid map (see below) and displays it in the item's page (Figure 2). When displaying a plasmid page, MyLabStocks also performs a BLAST query search for homologies to oligonucleotides and plasmid features. This way, users instantly have access to the list of oligonucleotides matching the plasmid, and to the coordinates of the features displayed on the map.

**Table 1:** List of major fields of records

	Fields
Plasmids	ID, Name, Link*, Other name, Sequence**, Author, Type, Marker 1, Marker 2, Description, Tags, Reporter, Promoter, Parent Vector, Insert, Insert Type, Reference, Date, Checkings, Bacterial selection, –20 Freezer, –80 Freezer, Fridge, Room
Oligonucleotides	ID, Sequence, Date, Author, Description, PCR conditions predicted, Purif, –20 Freezer, –80 Freezer, Fridge, Room
Antibodies	ID, Antigen, Product ID, Host Species, Type, Ordered by, Date, WB-dilution, Supplier, BatchRef, Freezer Location, Comments, –20 Freezer, –80 Freezer, Fridge, Room
Yeast strains	ID, Name, Other Name, Comments, General Background, Mating Type, ADE2, HIS3, … URA3, Locus1, …Locus 5, Parental Strain, Obtained by, Checkings, Cytoplasmic Character, Extrachromosomal Plasmid, Reference, Date, Author, –20 Freezer, –80 Freezer, Fridge, Room
Pipettes	ID, Serial number, Brand, Type
Pipettes history	ID, Date, Type of Event, Pipette's Serial Number, Usage after Event, Owner after Event, Comments
Laboratory notebooks	ID, Author, Serial number, Begin Date, End Date

* Name of sequence file uploaded in GENBANK format.

** Non-editable, directly loaded from the sequence file.

### Tables of annotations

Several fields of information can be selected from drop-down menus. This is the case for authors (laboratory members), alleles of yeast genes, the type of purification made on an oligonucleotide before it was stored, brands of pipets, storage location and several other fields. These menus are produced by MyLabStocks from tables of annotations, which can be edited with administrative privileges.

### Plasmid maps

Every time a plasmid page is viewed, MyLabStocks dynamically generates a plasmid map that is displayed on the page. This map is produced by PlasMapper (Dong *et al.*, 2004), which interrogates a database of plasmid features and draws a map displaying the features that match the plasmid sequence. Users can update the plasmid features database directly from within MyLabStocks, by editing a table of features the same way they edit tables of materials. Note that MyLabStocks displays plasmid maps only to facilitate database browsing and information retrieval. It is not dedicated to the detailed analysis of plasmid sequences. Other programs provide extensive possibilities for such analysis and are available either commercially or as open source. To perform plasmid sequence analysis, users can download the plasmid sequence from MyLabStocks and enter it in any of these programs.

### Searching the database using keywords and annotations

**Figure 3:**
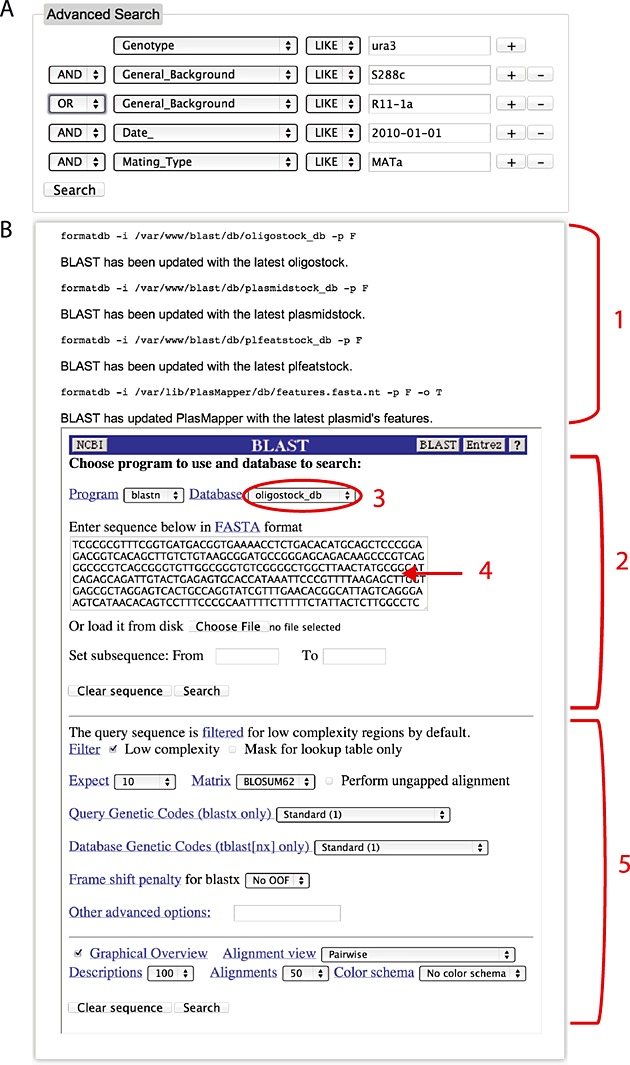
Searching information stored in MyLabStocks. (A) Records can be extracted using advanced search criteria on fields of annotations. (B) The database of MyLabStocks can also be searched using query sequences. Every time this page is accessed, BLAST databases are updated from the sequences of plasmids, oligonucleotides and plasmid features, respectively. The status (success or error) of this update is displayed in the upper area (1). A web interface of BLAST follows in area (2). The user can select from a dropdown menu (3) which database is to be searched (plasmids, oligos or features). The query sequence is entered in (4) or by uploading a sequence file. Optional parameters for the BLAST search can be selected from area (5)

The database can be searched in two ways. For easy and fast queries, a search option is directly available from every table of item. Depending on its nature, a field can be searched from queries entered in a text box or by selecting a value from a drop-down menu. If a search interrogates multiple fields, it returns the records matching all queries. MyLabStocks also provides a more advanced search option, where users can enter keywords in any number of fields, with OR and AND combinations (Figure 3A). For yeast strains, this advanced option allows to search the global genotype instead of one genetic locus at a time.

### Searching the database using BLAST

MyLabStocks is directly connected to a local BLAST server (Altschul *et al.*, 1990). Users can search oligonucleotides, plasmids or plasmid features simply by entering a query sequence and selecting a drop down menu in the BLAST interface (Figure 3B). Results are displayed within MyLabStocks in a standard BLAST output format.

### Connection and privileges

Users can connect to the database in any of four modes: view, add, edit and admin. The view mode allows users to freely navigate in any table and retrieve information without modifying it. The add mode allows user to add a record to a table. A single table can be accessed in this mode. For example, a specific login is necessary to access the table of plasmids in add mode, and this login does not provide access to the table of oligonucleotides or strains. The same applies to the edit mode, which allows to add and modify records. Preventing navigation across tables in add and edit modes minimizes the risk that users modify tables they did not intend to edit when they logged in. In addition, MyLabStocks displays a header band with a specific colour and warning message, inviting users to log out after their editing. The admin mode is provided to the super-user of the database (e.g. the principal investigator of the laboratory), who has all privileges on the tables. This allows him/her to perform frequent modifications of the database, such as adding a new laboratory member. Advanced administration of the database can be done with more complete tools, such as phpMyAdmin.

### Implementation

MyLabStocks is written in PHP/MySQL and is based on phpMyEdit (http://www.phpmyedit.org/). It can be used simultaneously by any number of clients using web browsers. Its sequence-based functionalities are performed by dynamic queries to BLAST (Altschul *et al.*, 1990) and PlasMapper (Dong *et al.*, 2004). These two programs are free and must be installed with MyLabStocks. The database is structured in tables of materials (such as plasmids, strains, primers or pipettes), tables of annotations that generate dropdown menus (such as laboratory members or commonly used alleles), and a table of the sequence features that users wish to display on plasmid maps. Users can connect in four different modes, each one allowing distinct privileges on specific tables.

### Back-ups

All users can back up the content of MyLabStocks by clicking on one of two links proposed on the home page. The first link offers a full back-up option, producing an archive containing all the MySQL tables stored in MyLabStocks, together with all files stored in the items' subdirectories. The other link offers to back up the MySQL tables only, without downloading all the subdirectories which may contain voluminous files. These archives can be stored on the local computer of the user, who can then copy them on any safe storage device or system. The documentation explains how to restore the database from back-ups.

### Further extensions

Extending and adapting MyLabStocks to specific needs of a laboratory is entirely possible with basic knowledge of MySQL. New tables can be created to handle other types of stocks (such as cell lines, nematodes or flies). New tables of annotations can also be created. The user interface is based on phpMyEdit, for which documentation is embedded in MyLabStocks. Changing phpMyEdit parameters allows the display of the newly created tables to be customized and unused ones to be hidden.

### Availability and requirements

MyLabStocks is freely available at: https://forge.cbp.ens-lyon.fr/redmine/projects/mylabstocks under an open source licence CeCILL V2.1. It can be installed on any operating system housing Apache, PHP, MySQL and Tomcat. A preinstalled virtual machine disk image is available upon request.
